# Mitral Valve Transcatheter Edge-to-Edge Repair Performed Exclusively with 3-Dimensional Intracardiac Echocardiography and Moderate Sedation

**DOI:** 10.1016/j.jscai.2022.100537

**Published:** 2022-11-25

**Authors:** Scott J. Hoffman, Pawan K. Hari, Paul J. Sarcia, Chris J. Reiff, Andrew J. Smith, Soma Sen

**Affiliations:** Division of Cardiology, Park Nicollet Methodist Hospital, St. Louis Park, Minnesota

**Keywords:** 3D-ICE, intracardiac echocardiography, MitraClip, moderate sedation, transcatheter edge-to-edge repair

Transcatheter valvular interventions provide symptomatic patients with minimally invasive treatment options, often without the need for general anesthesia (GA) or transesophageal echocardiography (TEE). However, mitral transcatheter edge-to-edge repair (TEER) is still performed almost exclusively with GA and TEE, requiring both endotracheal and esophageal intubation, which increase procedural risk and may be associated with poor outcomes. It was thought that the advent of 3-dimensional (3D) intracardiac echocardiography (ICE) could obviate the need for GA and TEE during mitral TEER; however, early-generation 3D-ICE catheters underperformed.

Recently, the VeriSight Pro (Philips) 3D-ICE catheter, containing an advanced imaging matrix with digital steering and multiplanar imaging capabilities, has been shown to produce high-quality images during structural heart interventions.^1^^,^^2^ Moreover, its modest profile and atraumatic design allow for uncomplicated crossing of the interatrial septum after transseptal puncture (TSP) without the need for a second TSP or balloon atrial septostomy, ideal for interventions performed in the left atrium (LA), including mitral TEER.

## Case report

An 87-year-old woman with moderate-to-severe primary mitral regurgitation (MR) and dyspnea was evaluated for TEER. Preprocedural TEE could not be completed because of a severe esophageal stenosis. After a heart team discussion, the patient elected to proceed with mitral TEER using 3D-ICE for imaging guidance.

**Figure 1:**
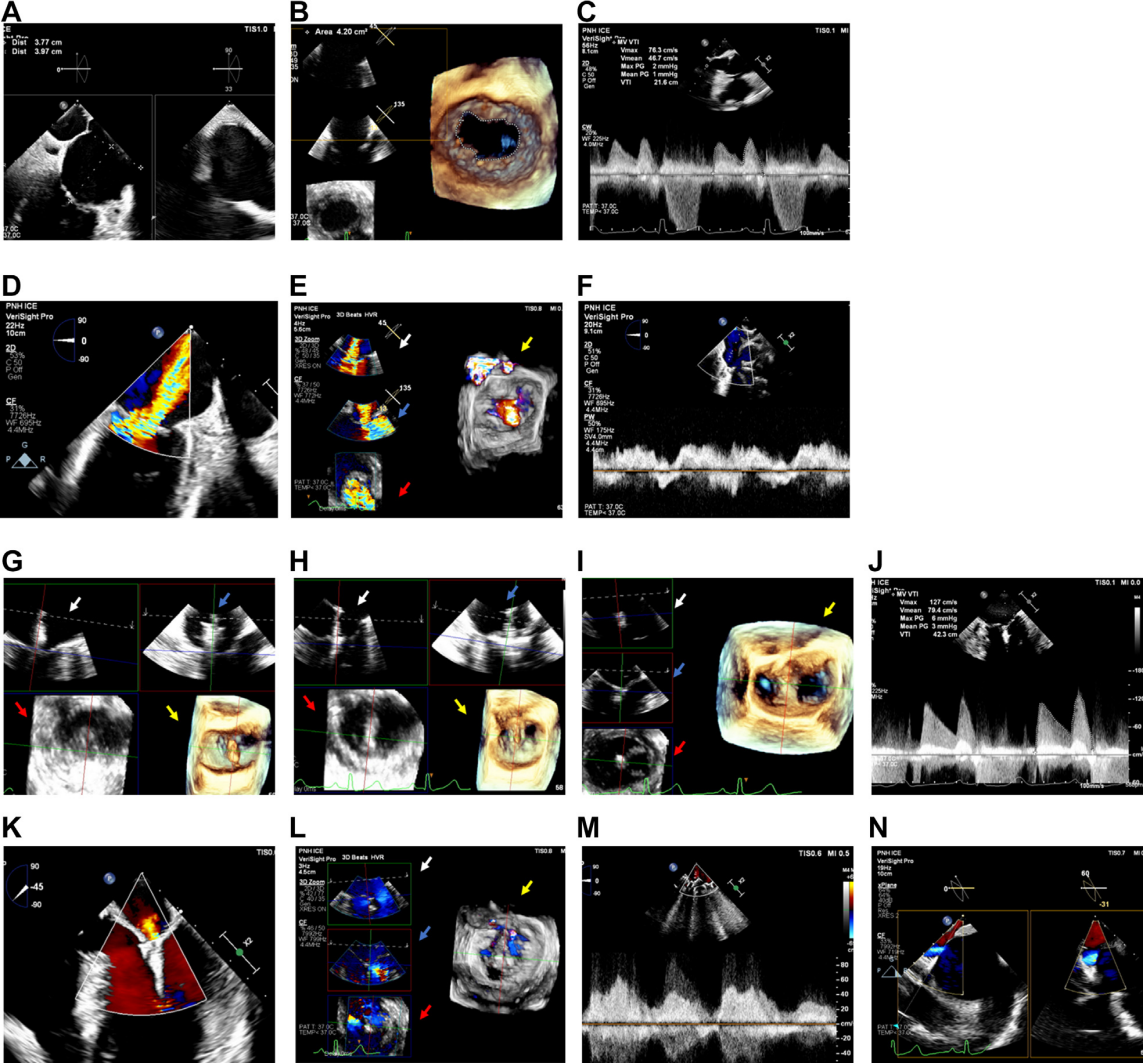
**Mitral valve transcatheter edge-to-edge repair using 3-dimensionalintracardiacechocardiography** (**A**) Transseptal puncture followed by detailed mitral valve (MV) evaluation using 3-dimensional (3D) intracardiac echocardiography, including (**B**) area by 3D planimetry, (**C**) mean gradient, as well as (**D**) mitral regurgitation assessment by 2-dimensional (2D) color imaging, (**E**) multiplanar reconstruction with color (bicommissural [white arrow], left ventricular outflow tract [blue arrow], en face 2D [red arrow], and en face 3D views [yellow arrow]), and (**F**) pulmonary vein Doppler analysis, revealing systolic blunting. Multiplanar imaging of the MitraClip in the (**G**) left atrium, (**H**) left ventricle, and (**I**) grasping the MV leaflets. (**J**) The final mean MV gradient. (**K**) 2D and (**L**) multiplanar views of MV leaflet insertion, with reduced mitral regurgitation. (**M**) Pulmonary vein Doppler, revealing systolic dominant flow. (**N**) 2D color X-plane image of residual interatrial shunt.

Moderate sedation was administered rather than GA to reduce the risk of periprocedural aspiration given the patient’s underlying dysphagia. Bilateral femoral venous access was obtained, including placement of a right-sided 16F sheath and a left-sided 10F sheath. A 9F VeriSight Pro 3D-ICE catheter was introduced through the 10F sheath and advanced into the right atrium to identify a TSP site in the fossa ovalis, providing adequate height to the mitral commissural line (Figure 1A). A Preface sheath and Heartspan needle (Biosense Webster) were used to perform a standard TSP, facilitating the placement of an extra small Safari wire (Boston Scientific) in the LA. A MitraClip guide (Abbott) was advanced over the Safari wire and used to dilate the TSP site by successively moving it back and forth 3 times across the interatrial septum; then, the guide was withdrawn into the inferior vena cava. Under fluoroscopic and ICE guidance, the VeriSight Pro catheter was advanced across the dilated TSP site into the LA, and a pre-MitraClip assessment was performed, revealing adequate mitral valve (MV) area, a mean MV gradient of 1 mm Hg, and moderate-to-severe MR with blunting of systolic pulmonary vein flow (Figure 1B-F); thus, an NTW clip (Abbott) was selected.

The MitraClip guide was readvanced into the LA, followed by the NTW device. Multiplanar ICE imaging was used to guide the clip toward the MR, advance it into the left ventricle, and grasp the MV leaflets (Figures 1G-I and Supplemental Video 1). After a thorough ICE evaluation, the clip was released, leaving a mean MV gradient of 3 mm Hg, trace MR, and normal systolic pulmonary vein flow (Figure 1J-M). A small left-to-right interatrial shunt was detected (Figure 1N). There were no complications. The total procedure time, from vascular access to closure, was 112 minutes. The patient was discharged the next day after transthoracic echocardiography confirmed trace MR and a mean MV gradient of 2 mm Hg.

## Discussion

To our knowledge, this is the first report demonstrating TEER with MitraClip using the VeriSight Pro 3D-ICE catheter as the exclusive echo imaging modality with moderate sedation. Previous reports have involved simultaneous TEE and 3D-ICE imaging, GA, and/or a bulkier 3D-ICE catheter, the latter of which required large-bore venous access and atrial septostomy to enter the LA.^2^, ^3^, ^4^

In this case, 3D-ICE delivered a wide range of high-quality mitral images required for TEER, primarily through digital steering, which is not always feasible with TEE because of various anatomical constraints or acoustic shadowing from the MitraClip delivery system while imaging from the esophagus. Rarely, VeriSight catheter manipulation was required to overcome shadowing artifact or maintain essential MV windows, which were infrequently disrupted by catheter interaction with the MitraClip guide through the lone TSP site. However, restricted far-field imaging, related to limited crystal elements in the imaging matrix of the VeriSight Pro, did inhibit real-time assessment of the MitraClip guide position across the interatrial septum. Thus, it may be advisable to avoid 3D-ICE as the sole imaging modality to guide mitral TEER if challenging atrial septal anatomy is identified (eg, lipomatous septum or presence of a septal closure device); however, fluoroscopy could be a useful imaging adjunct in this setting.

## Conclusion

This case demonstrates a minimalist approach to mitral TEER using 3D-ICE guidance and moderate sedation, with the potential to reduce procedural risk and accelerate recovery time for numerous patients.

## Supplementary material

Supplemental Video 1 Caption

Video 1 Grasp of the mitral valve leaflets with a MitraClip in the left ventricle under 3-dimensional intracardiac echocardiography guidance with multiplanar reconstruction, including bicommissural, left ventricular outflow tract, en face 2-dimensional, and en face 3-dimensional views.
