# Supplementary material for: Mitral Valve Transcatheter Edge-to-Edge Repair Performed Exclusively with 3-Dimensional Intracardiac Echocardiography and Moderate Sedation
Source: J Soc Cardiovasc Angiogr Interv. 2022 Nov 25;2(1):100537. doi: 10.1016/j.jscai.2022.100537 (PMC11308044; doi:10.1016/j.jscai.2022.100537)
Supplement: Supplemental Video 1 Caption [file mmc1.docx]

**SUPPLEMENTARY VIDEO CAPTION**

**SUPPLEMENTARY VIDEO 1 –** Grasp of the Mitral Valve (MV) leaflets with a MitraClip in the left ventricle, under 3D-ICE guidance with multiplanar reconstruction, including bicommissural, left ventricular outflow tract, en face 2D, and en face 3D views.
